# Freshwater Lichens, Including New Species in the Genera *Verrucaria, Placopyrenium* and *Circinaria*, Associated with *Lobothallia hydrocharis* (Poelt & Nimis) Sohrabi & Nimis from Watercourses of Sardinia

**DOI:** 10.3390/jof9030380

**Published:** 2023-03-21

**Authors:** Juri Nascimbene, Pier Luigi Nimis, Johanna Klüßendorf, Holger Thüs

**Affiliations:** 1 Biome-Lab, Department of Biological, Geological and Environmental Sciences, Alma Mater Studiorum, University of Bologna, Via Irnerio 42, I-40126 Bologna, Italy; 2 Department of Life Sciences, University of Trieste, Via Giorgieri 10, I-34127 Trieste, Italy; 3 Department of Botany, State Museum of Natural History Stuttgart, Rosenstein 1, D-270191 Stuttgart, Germany

**Keywords:** biogeography, cryptic diversity, DNA-barcoding, Mediterranean, taxonomy

## Abstract

This work summarizes the results of the exploration of freshwater lichen biota on the island of Sardinia associated with the regional flagship species *Lobothallia hydrocharis*, a large-sized crustose lichen from the splash zone along mountain streams, so far known from Sardinia only. Molecular data were used to confirm its distinctiveness from other taxa and its systematic placement and to identify critical taxa among its associated lichen biota. We found 25 species of lichenized fungi, including three species new to science in the genera *Verrucaria, Placopyrenium*, and *Circinaria*, and seven species new to Sardinia (*Hydropunctaria rheithrophila*, *Ionaspis chrysophana*, *I. odora*, *Verrucaria aquatilis*, *V. collematodes*, *V. pseudovirescens*), or new to Southern Europe (*V. devensis*). Specific traits for the freshwater lichen biota of Sardinia were identified and compared to those reported from freshwater sites in the Alps and Carpathian mountains, e.g., a relative scarcity of subgelatinous lichens. Parasitic or epilichenic interactions were found frequently but only in the splash zone and not in the permanently submerged zone, i.e., two parasitic *Placopyrenium* species, and clearly lichenicolous thalli of *Kuettlingeria atroflava* and *Lobothallia hydrocharis*. Due to its specific trait profile and the great potential for the discovery of new species, we recommend the inclusion of Sardinian and further Mediterranean sites in continental-scale monitoring programs for freshwater lichens.

## 1. Introduction

Lichenized fungi have the largest diversity in terrestrial habitats, while relatively few taxa exist in permanently or temporarily inundated sites in and along watercourses [1], where they have to cope with several limiting factors, such as excessive hydration over long periods, repeated submersion/emersion cycles, substrate instability, and strong currents [2]. These pressures resulted in the evolution of convergent morphological adaptations even across distantly related taxa, thus making species identification difficult [3].

In recent decades, the freshwater lichen biota of northern and central Europe has been being increasingly studied [4,5,6,7,8], shedding light both on phylogenetic relationships within critical groups (such as the family Verrucariaceae) [9,10,11,12] and on the ecological requirements of the species [13,14,15]. In contrast, for Mediterranean areas, field inventories are scarce [16,17,18] and molecular data complementing morphologically identified material of freshwater lichens are missing. Major gaps in the knowledge of species distribution and ecology remain, and we expect that new freshwater lichens are likely to be discovered in this region.

The peculiarity of Mediterranean freshwater lichen biota is corroborated by the emblematic case of a flagship species, *Lobothallia hydrocharis* (Poelt & Nimis) Sohrabi & Nimis, that until now was known only from the splash water zone of a mountain stream in Sardinia, where it is locally dominant. It is a large-sized placodioid lichen described on the basis of a collection from the province of Nuoro [16]. Since then, no one has ever investigated the pool of species associated with this lichen. Moreover, its phylogenetic position in the *Lobothallia* clade was never tested by a molecular approach. *Lobothallia* is a morphologically and genetically well-delimited genus within the Megasporaceae, a family which is represented with particularly high species numbers in dry habitats but also includes some characteristic representatives from freshwater habitats. They can occupy extensive areas and locally dominate the lichen community in the splash water zones, e.g., in mountain streams of Central Europe [19,20]. Species identification and sometimes even generic affiliation can be difficult due to the scarce development of diagnostical features (e.g., ascospores are seldomly formed in some species and pycnidia not always present [21] and the lack of reference sequences for many poorly known species that have not been collected in recent years.

In this work, we summarize the results of our exploration of the freshwater lichen biota of Sardinia associated with the regional flagship species *Lobothallia hydrocharis*, adding several new localities to its Sardinian distribution. In order to verify species-level delimitation and systematic placement of this characteristic lichen, we also studied its systematic placement among other species of *Lobothallia*. For the associated lichenized biota, we tried to complement as many morphological identifications as possible by DNA barcoding data from the ITS marker region, which contributed to the description of three species as new to science.

## 2. Materials and Methods

### 2.1. Field Work and Taxon Sampling

**Figure 1:**
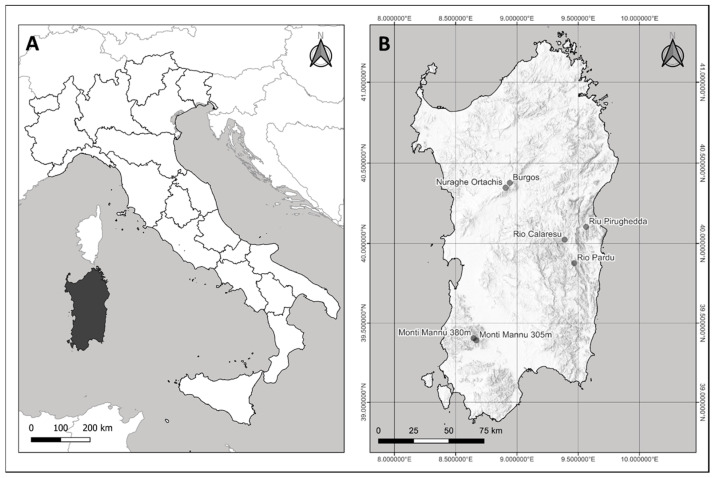
Geographic location of the island of Sardinia (in black) (**A**) and collection sites (**B**). The locality at Rio Pardu refers to the collecting site of a specimen from GZU, while the other localities refer to our survey.

**Figure 2:**
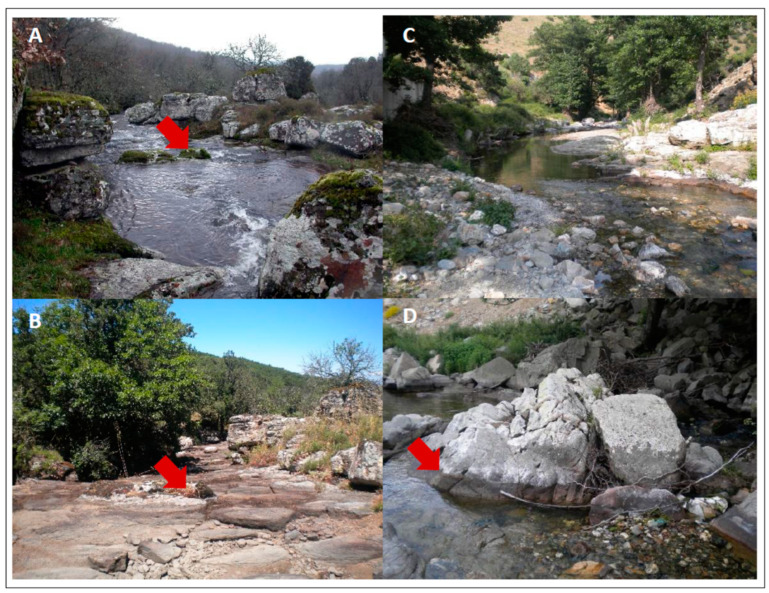
Habitats of freshwater lichens in Sardinia. (**A**,**B**) collection site of Nuraghe Ortachis-Badde Salighe in spring, with abundant water flow (**A**), and during summer drought when water is almost missing (**B**). Red arrows indicate reference points for comparison. (**C**) Collection site of Rio Calaresu during summer drought. (**D**) Lichen zonation at Rio Calaresu, well visible during summer drought. The dark zone near the water (the usually submerged zone) is marked by Verrucariaceae, and it is followed upward by a grey zone (the splash zone) that is mainly determined by Megasporaceae, including *Lobothallia hydrocharys*. The red arrow indicates the transition between the usually submerged and the splash zones.

Specimens were collected between 2009 and 2016 in watercourses characterized by the occurrence of the flagship species *Lobothallia hydrocharis,* mostly during a field campaign held in 2014 to assess its distribution and conservation status [22]. Collection sites are distributed across the main mountain areas of Sardinia (Figure 1A,B) at elevations ranging from about 300 m (Montimannu) to about 1050 m (Badde Salighe), on siliceous bedrock. At these sites, the mean annual temperature ranges between 12 and 15 °C and precipitation between 700 and 1000 mmy^−1^ with a marked summer drought. Some sites are almost completely dry during the summer drought (Figure 2A,B), while others maintain a small water flow also in summer (Figure 2C), making evident a typical lichen zoning along the water gradient (Figure 2D).

In addition, vouchers corresponding to previous records of freshwater lichens from Sardinia [16] from the collection at the university of Graz (GZU) were re-examined. Nomenclature, as well as synonyms for old records, mainly follow ITALIC 7.0—the information system on Italian lichens [23]. This source was also used for retrieving information on species’ ecological and distributional patterns in Italy. For each taxon, the following information is included: (a) province, collection locality, coordinates (WGS 84) elevation, and substrate; (b) collection date; (c) collector(s) name (Leg.), and Herbarium code. Institutional herbaria are abbreviated according to Index Herbariorum [24].

Most vouchers are deposited either at the State Museum of Natural History Stuttgart (STU) or in the private herbarium of J. Nascimbene. Specimens from the latter private herbarium, which is abbreviated as “Herb. Nascimbene”, are incorporated in the collection of the University of Bologna (BOLO); (d) short notes on ecology, distribution and/or taxonomy.

### 2.2. Morphological Studies and Metabolite Reactions

Anatomical characters were studied on hand sections and microtome sections prepared with a cryotome (SLEE, Mainz, Germany) with a section thickness of 15 µm. All measurements were carried out in tap water using a Zeiss Axioscope (brightfield illumination) or Olympus BX53 with Differential Interference Contrast. Spore measurements are given as [minimum]–[median]–[maximum], followed by the number of observations. Tests for reaction of medullary and hymenium tissue with Lugol’s Iodine were checked on sections under the microscope, and Apothecial pigments were treated with 50% HNO_3_, 15% HCL and 20% KOH solutions following the protocol by [25]. Reactions with KOH were also studied on thallus fragments under a dissecting microscope, and tests with sodium hypochlorite- and p-phenylenediamine solutions were prepared according to [26] in the same way. Acetone-soluble metabolites were visualized by the standard protocol for HPTLC with solvent systems A (Toluene:dioxane:acetic acid, 45:15:2), B (Cyclo-hexane:methyl tertiary-butyl ether:formic acid, 6-5:5:1) and C (Toluene:acetic acid, 20:3) [26].

### 2.3. DNA-Barcoding

For DNA-extraction, the Nucleo-Spin Plant Kit (Machery Nagel, Düren, Germany) and Quiamp Investigator Kit (Qiagen, Hilden, Germany) were used with overnight lysis of samples. Amplification of the ITS barcode marker was carried out with Illustra Puretaq PCR Beads and MyTaqRed-Mix (Bioline, Heidelberg, Germany) using primers ITS1F, ITS4 and LR3 following the protocols by [11]. We tried first to obtain a longer fragment with primer combination ITS1F/LR3, and only if this failed, amplification of the shorter fragment with the primer combination ITS1F/ITS4 was carried out. Attempts to amplify nuLSU and mtSSU with the primers LROR/LR7, LROR/LR6 and mrssu1/MSU7 following the protocols by [27] did not produce PCR-products.

### 2.4. Phylogenetic Analyses

Sequencing reads were manually proof-checked and low-quality parts at the start and end of the reads removed. Reads were further checked by comparing forward and reverse reads and remaining ambiguities coded accordingly.

All new sequences were subjected to an initial exploratory BLASTn-search against available sequences on the GenBank nucleotide collection [28]. Percentage identities > 98% were considered as near identical.

For three putative new *Circinaria* and *Aspicilia* species and one *Verrucaria* sp., we found only low similarity matches (94% and lower) with currently described species. Only *Circinaria ochracea* sp. nov. is represented by multiple collections and sequences. For this taxon, a subsequent exploratory analysis with a selection of ITS sequence data of named *Circinaria* species was carried out, but the resulting ITS genetree-topology has low support values for most of the basal branches and should be treated with caution. For this reason, we have provided its topology only in the Supplementary Materials (Supplementary Materials Figure S1). 

For the analysis of the placement of *Lobothallia hydrocharis* within the genus *Lobothallia* two representative sequences from GenBank for each of the species included in recent taxonomic treatments of *Lobothallia* species [29,30] were chosen. 

The selection of reference sequences and phylogenetic analyses for Verrucariaceae followed the procedures by [31], e.g., for the final alignment, was based on the results of an initial BLAST-search against available GenBank Sequences with a threshold of at least 90% identity. Further sequences were included that represent type specimens of taxa with morphological traits that overlap with features of the new species. 

Voucher information for the specimens that are represented by sequences from GenBank is provided in the Supplementary Materials (Supplementary Materials Table S1). 

Sequences were aligned by MAFFT and ambiguous regions marked using the GUIDANCE2-server (http://guidance.tau.ac.il/ver2/, accessed on 1 November 2022), followed by manual checks and adjustments in BioEdit 7.2.6.1 [32], e.g., ambiguous regions (positions with a confidence score below 0.93) and flanking parts of the 18S and 28S rDNA from GenBank reference sequences that were not covered in our newly generated ITS sequences were removed prior to subsequent analysis. Likelihood models were selected using the Bayesian Information Criterion (BIC) as implemented in jModelTest [33] and ModelFinder [34]. Maximum Likelihood-based analyses were carried out with RAxML8.2 [35], operated via raxmlGUI2.0 [36], and with IQ-TREE [37] on the IQ-TREE webserver (vhttp://iqtree.cibiv.univie.ac.at, accessed on 1 November 2022) with model selection based on [34] and ultrafast bootstrapping for estimating node supports [38]. Bayesian inference of phylogeny was carried out by Markov Chain Monte Carlo sampling as implemented in MrBayes.v3.3.6 [39] using the models with the closest fit to those suggested by jmodeltest [34] with two independent chains and 1.000.000 generations from which every 100th was sampled. The first 25% of the sampled trees was discarded as *burnin*. Phylogenetic trees were visualized by FigTree (http://tree.bio.ed.ac.uk/software/figtree/, (accessed on 1 November 2022)), and final editing was carried out in Inkscape [40].

### 2.5. Comparison with Freshwater Lichen Biota under Different Bioclimatic Conditions

To qualitatively evaluate the potential peculiarity, in terms of species composition and traits, of the freshwater lichen biota of our Sardina survey, we assembled a dataset including two comparable surveys from mountain areas under different bioclimatic ranges: (1) the Alps—we extracted the list of freshwater lichens from the recent checklist of the Paneveggio-Pale di San Martino Natural Park [41]. This is a typical landscape of the Alps spanning an elevational gradient of about 2000 m (range 1200–3200 m) where annual precipitation ranges between 1100 and 1600 mm and mean annual temperature varies between 8 and −1 °C, depending on elevation; (2) Eastern Carpathian Mts.—we extracted the list of freshwater lichens from [42], including the submerged and splash zones. This is a mid-sized mountain chain in a Central-European context with elevation slightly exceeding 1400 m, annual rainfall of about 1000–1300 mm and mean annual temperature between 4 and 5 °C. For all the species, we retrieved information from ITALIC 7.0—the information system on Italian Lichens [23] on selected traits that can be indicative of adaptation to different bioclimatic conditions [43]: thallus growth form (crustose, foliose, fruticose), photobiont type (Chlorophyta other than Trentepohliaceae, Trentepohliaceae, Cyanobacteria), gelatinous/nongelatinous thallus, and subgelatinous/nonsubgelatinous thallus.

## 3. Results

We obtained 16 new ITS sequences of 10 different taxa (Table 1). Attempts to amplify further specimens and additional markers (nuLSU, mtSSU) failed. Amplicon quality varied greatly and was linked to the time period since collection and the humidity of the micro-habitat. None of the collections from 2010 produced amplicons. For the collections from 2014, all collections from fully submerged sites failed. The success rate for specimens from the splash water zone was higher, but extractions of many specimens still did not produce PCR products.

For Verrucariaceae, sequence data after the removal of ambiguously aligned positions in the final alignment consisted of 543 sites with 319 constant sites, 195 parsimony informative sites and 271 distinct site patterns. *Placopyrenium* was retrieved as a monophyletic group with *P. pseudocinereoatrum* Thüs & Nascimbene sp. nov. placed in the basal position to a well-supported clade with four collections of *Placopyrenium cinereoatrum* (Degel.) Orange from the British Isles, Finland and the Czech Republic. Our collection of *Verrucaria ruderum* DC is nested within *V. viridula* s.lat., and *V. mediterranea* Thüs & Nascimbene sp. nov. is placed in a clade with the informal taxon “*Verrucaria* sp. 10” and the recently described *V. tenuispora* Vondrák & Thüs.

**Figure 3:**
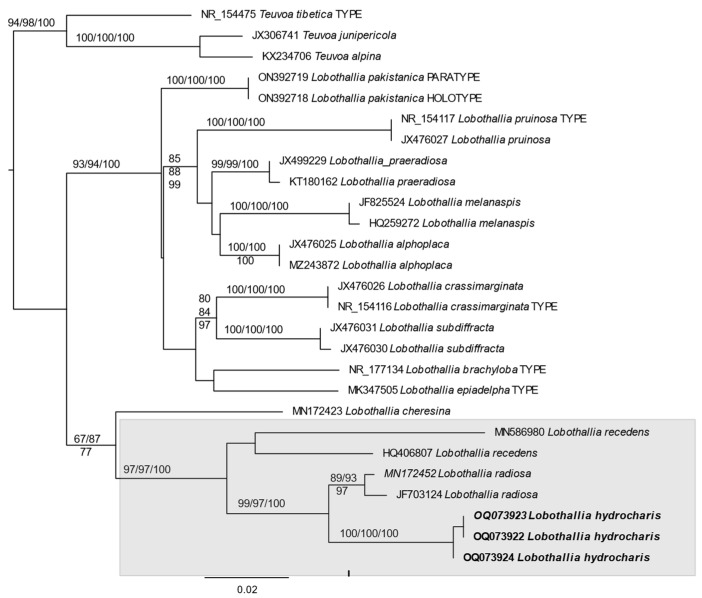
Topology of best phylogenetic RAxML tree based on ITS data of *Lobothallia*, with *Teuvoa* as the outgroup taxon. The numbers in each node represent bootstrap support (BS) from RAxML analyses, ultrafast bootstrap support from IQTree-analysis, and posterior probability (PP) values from Bayesian Inference of phylogeny. Only BS values ≥ 75% and PP values ≥ 0.95 were plotted on the branches of the tree. GenBank Accession Numbers are given before each taxon name, Sardinian collections are marked in bold.

*Lobothallia* is retrieved as monophyletic in our ITS-genetree with *Teuva* as the outgroup. Within *Lobothallia*, *L. hydrocharis* is placed with high support as a sister species to *L. radiosa* (Hoffm.) Hafellner and together with that species in the same clade as *L. recedens* (Taylor) A. Nordin, Savić & Tibell (Figure 3).

BLAST-searches on GenBank for similar sequences of other Megasporacerae to those that we obtained from our Sardinian *Circinaria ochracea* Thüs & Nascimbene sp. nov., *Aspicilia* sp. 1 and *Aspicilia* sp. 2 have resulted in low identities (<95%) with existing sequences, but all closest matches are crustose Megasporaceae. The placement of the new sequences in an ITS-genetree of the family Megasporaceae remained inconclusive due to the short length of the amplified ITS-fragments, which resulted in a high frequency of ambiguities in the alignment at the family level and a poor resolution of the genetree (ITS-genetree in Supplementary Materials Figure S1).

**Figure 4:**
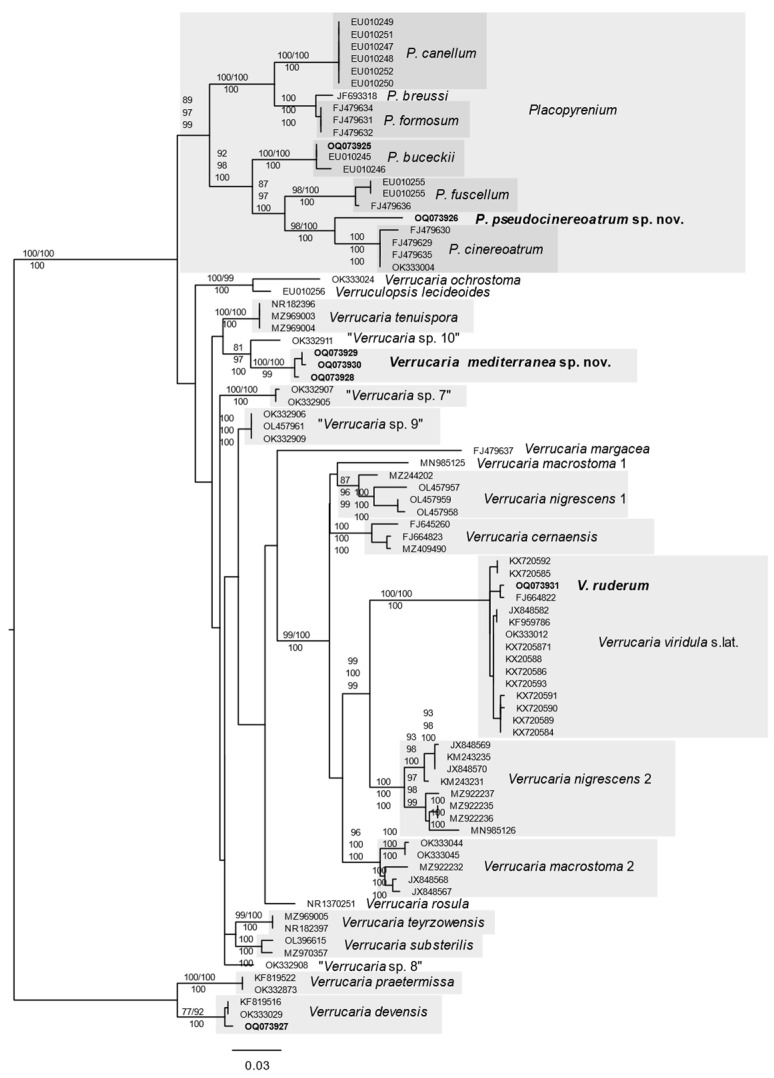
Topology of best phylogenetic RAxML tree based on ITS data of *Verrucariaceae*, with *Verrucaria devensis* and *V. praetermissa* as outgroup. The numbers in each node represent bootstrap support (BS) from RAxML analyses, ultrafast bootstrap support from IQTree-analysis and posterior probability (PP) values from Bayesian Inference of phylogeny. Only BS values ≥ 75% and PP values ≥ 0.95 were plotted on the branches of the tree. GenBank accession numbers are given before each taxon name, and Sardinian collections are marked in bold.

In the Verrucariaceae-ITS-genetree (Figure 4) *Placopyrenium pseudocinereoatrum* sp. nov. is placed as sister taxon in a basal position to *P. cinereoatrum.* The sequence of our Sardinian collection of *P. bucekii* (Nádv. & Servít) Breuss is near identical with the one for a French collection of this species and placed basal to the clade of *P. fuscellum* (Turner) Gueidan & Cl. Roux., *P. pseudocinereoatrum* sp. nov. and *P. cinereoatrum*. *Verrucaria ruderum* is nested within *V. viridula* s. lat. and *V. mediterranea* sp. nov. placed in a clade together with “*Verrucaria* sp. 10” from the Czech Republic, an informal taxon related to *V. tenuispora* [44]. Older sequences from Genbank for specimens named as *V. nigrescens* Pers. and *V. macrostoma* DC. are placed in different clades (“*V. nigrescens* 1”, “*V. nigrescens* 2”, and “*V. macrostoma* 1”, “*V. marcostoma* 2”) and all of them are placed in one clade together with *V. viridula* s. lat., but none of them is closely related to *V. mediterranea* sp. nov.

### 3.1. Freshwater Lichens in Sardinian Streams

***Aspicilia aquatica* (Fr.) Körb.**

Oristano Prov., Marghine mountain chain, Nuraghe Ortachis, Badde Salighe; N40.347791 E8.906399; 1030 m; July 2014; Nascimbene leg.; on siliceous rock, in the splash zone; BOLO-Herb. Nascimbene 4855 (overgrown by *Lobothallia hydrocharis*), SMNS-STU-F-0001655 (STU).

This sub-aquatic species had been recorded before from two other sites on Sardinia [16], both from the NE province of Nuoro. The new collection is the first one from the province of Oristano in the W of the island, the species apparently being a common associate of *Lobothallia hydrocharis*.

***Aspicilia* sp. 1**

Carbonia-Iglesias Prov., Domus Novas, Duchessa, Monti Mannu; N39.392102 E8.669081; 305 m; July 2014; Nascimbene leg.; SMNS-STU-F-0002802 (STU).

**Figure 5:**
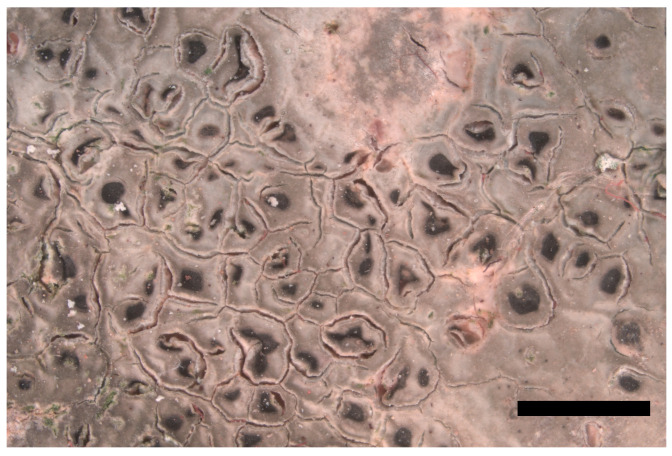
Thallus of *Aspicilia* sp. 1, SMNS-STU-F0002802. Scale bar = 2 mm.

**Description:** *prothallus* white thin. *Thallus* crustose, episubstratic, up to 120 µm high, composed of light grey to light greenish olive areoles up, with a smooth, almost “oily” appearance (Figure 5). *Cortex* thick, 10–30 µm, mostly colorless, only the uppermost layer (app. 10 µm) with dark pigment, producing a faintly yellowish solution after application of 20% KOH. *Photobiont layer* varying from 15 to 60 µm high, cells with central chloroplast (trebouxioid), the cells up to 9–14 µm in diameter. *Medulla* white, 40–90 µm high, hydrophobic, with air-filled spaces in water-mounted sections. *Apothecia* lecanorine-aspicilioid (immersed in the thallus), disc shape rounded, up to 0.4 mm in diam., to elongated, app. 0.75 × 0.3 mm., exciple indistinct, hymenium ca. 95 µm high, not inspersed with oil droplets, I+ blue, upper hymenium with a greenish-yellowish-grey pigment in water. Yellowish pigment dissolving in HNO_3_, leaving the emerald green color of *Caesiocinera*-green pigment. Although asci are visible in sections of the samples, no spores could be detected. *Pycnidia* not seen. 

**Chemistry:** medulla K+ yellow slowly turning reddish, C-, I-, P-. HPTLC: Stictic acid (major) and norstictic acid (minor). 

**Habitat and Distribution:** found only once in the splash water zone of a stream on siliceous rock.

**Notes:** Based on its rather thin and smooth thallus, the greyish-olive color, and its chemical traits, we identified the specimen first as *A. laevata* (Ach.) Arnold, but ITS data of the Sardinian specimen have little similarity with reference sequences of this species, suggesting that it may represent another possibly undescribed species. Morphologically the Sardinian lichen differs from *A. laevata* by the poorly differentiated exciple and fully immersed apothecia.

***Aspicilia* sp. 2**

Sassari Prov.; Burgos; N40.37795 E8.941887; 820 m; July 2014; Nascimbene leg.; on siliceous rock, in the splash zone; SMNS-STU-F-0002796 (STU). 

**Figure 6:**
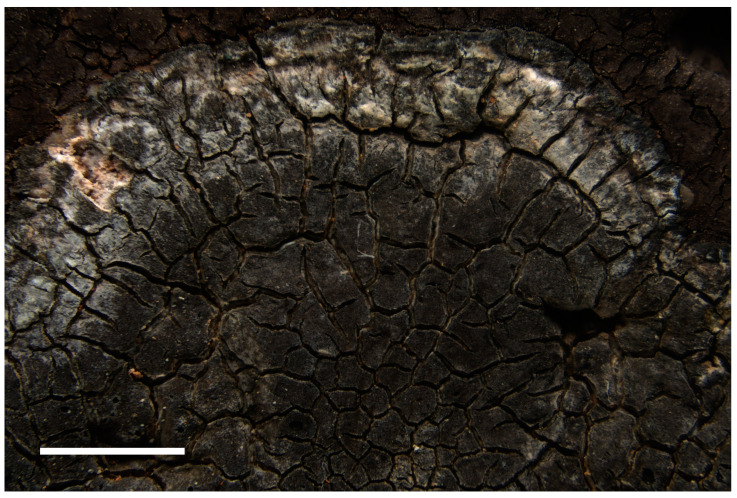
Thallus of *Aspicilia* sp. 2, SMNS-STU-F0002796. Scale bar = 0.5 mm.

**Description:** *thallus* crustose, episubstratic, up to 240 µm high, composed of flat, dark grey areoles up to 1.0–1.2 mm in diam., surrounded by a whitish prothallus (Figure 6). *Cortex* of 2–3 cell layers, only the uppermost layer with dark pigment, producing a faintly yellowish solution after application of 20% KOH. *Photobiont layer* 80–90 µm high, photobionts with central chloroplast (trebouxioid), the cells up to 9–11 µm in diameter. *Medulla* white, 90–100 µm high, hydrophobic, with air-filled spaces in water-mounted sections and mineral fragments in its lowermost parts, K-, C-, I-, PD+, orange. *Apothecia* lecanorine-aspicilioid (immersed in the thallus), disc shape rounded, not elongated, 0.4–0.7 mm in diam., exciple ca. 20 µm wide, hymenium ca. 180 µm high (incl. subhymenium 20 µm), inspersed with small oil droplets, I+ at first bluish but with increasing iodine-concentration quickly turning deep orange. Upper hymenium dark olive to brown-black, brown-black pigment dissolving in HNO_3_, leaving a faint greenish pigmentation with *Caesiocinera*-green characteristics. Although asci are visible in sections of the samples, no spores could be detected. *Pycnidia* not seen. 

**Chemistry:** stictic acid (major), stictic acid co-metabolite (minor).

**Habitat and Distribution**: found only once in the splash water zone of a stream on siliceous rock,

**Notes:** we obtained an ITS sequence from this species, which is clearly different from any named sequence from GenBank and also from *Circinaria ochracea* sp. nov. but similar, although not identical, to the one we obtained from *Aspicilia* sp. 1, which has a lighter, much thinner thallus with a shiny “oily” appearance, absent prothallus and different spot test reactions. In the absence of data for more conservative markers and the lack of ascospores and pycnidiospores in our material, we provisionally place this unknown (possibly new) species in the genus *Aspicilia* in a wide sense, but this placement and a possible formal description of these two taxa as new species should be reassessed when data on the shape of its ascospores and conidia and more conservative marker sequences from freshly collected additional specimens become available.

***Circinaria ochracea* Thüs & Nascimbene sp. nov. MB 847528**

Holotype: Sassari Prov.; Burgos; N40.37795 E8.941887; 820 m; July 2014; Nascimbene leg.; on siliceous rock, in the splash zone of a stream; -SMNS-STU-F-0002797 (STU); isotypes: BOLO-Herb. Nascimbene 7208, SMNS-STU-F-0002927 (STU) (host for *Placopyrenium pseudocinereoatrum* sp. nov. and filed under that name). 

**Etymology:** the species epithet “ochracea” refers to the ocher color of the thallus of the new species.

**Diagnosis:** similar to *A. cupreoglauca* and orange forms of *A. intermutans* but with a different chemistry: K- or K+ only faintly yellow (pigment “leaking” in the KOH-solution in direct vicinity of the thallus), and no metabolites visible by HPTLC. 

**Figure 7:**
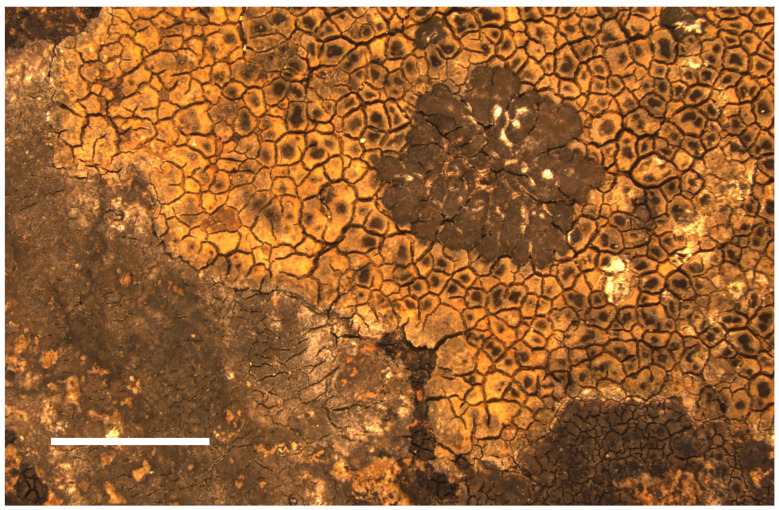
*Lobothallia hydrocharis* growing on the holotype specimen of *Circinaria ochracea* sp. nov., SMNS-STU-F0002797 (STU). Scale bar = 0.5 mm.

**Figure 8:**
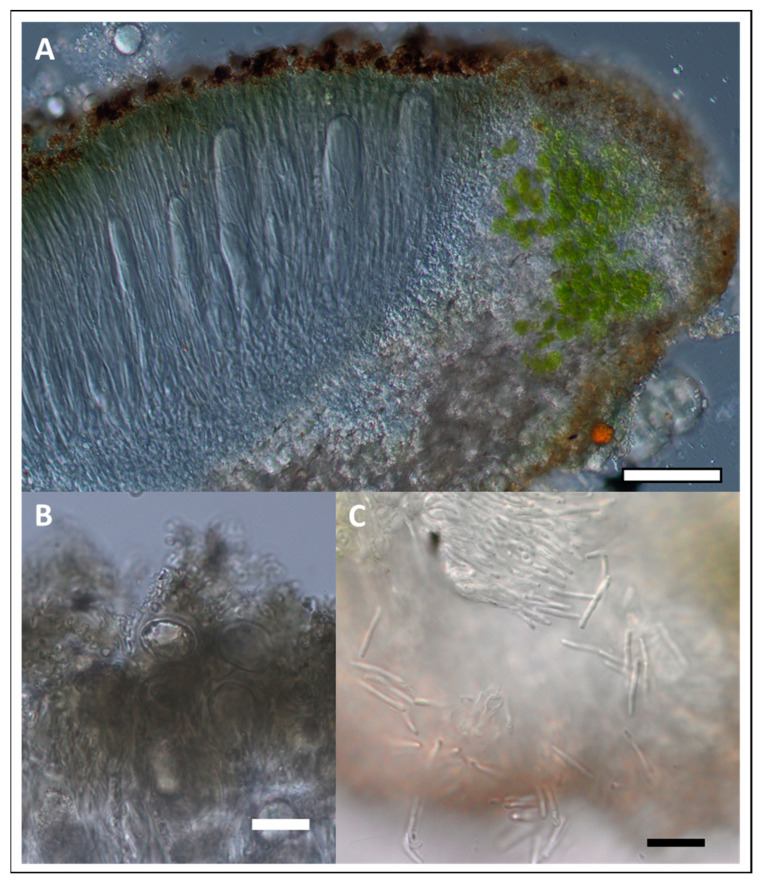
Holotype of *Circinaria ochracea*, SMNS-STU-F0002797 (STU). (**A**) Section of an apothecium. (**B**) Globose ascospores. Scale bar =2 0 µm. (**C**) Pycnospores. Scale bar = 10 µm.

**Description:** *prothallus* absent or colorless but marked by the accumulation of light-colored fine sediment particles, *thallus* crustose, episubstratic, 145–170 µm thick, composed of flat, yellow-orange 0.5–0.9 mm wide areoles (Figure 7). *Cortex* 7–8 µm high, with a yellowish-greenish-grey pigment. *Photobiont layer* 40–80 µm high, photobionts with central chloroplast (trebouxioid), cells up to 9–11 µm in diameter. *Medulla* white, 60–85 µm high, hydrophobic and with air-filled spaces in water-mounted microscopic sections. *Thallus* (medulla) K-, but cortex pigment partly dissolving in 20% KOH solution creating a faintly yellow color in the KOH- drop in the direct vicinity of thallus fragments. No reactions with potassium hypochlorite (C) or p-phenylenediamine (P). *Apothecia* aspicilioid (immersed), disc shape rounded to elongated, 0.3–0.5 mm wide, excipulum 45–50 µm thick, hymenium (123–) 200–250 µm high, clear without oil droplets, paraphyses not moniliform, I+, bluish, uppermost part with *Caesiocinerea*-green, intensifying emerald green in HNO_3_, pale yellowish in KOH and weakly green in HCL, epihymenium ca. 25 µm high, with dark yellowish-brown pigment (Figure 8A), partly soluble in HNO_3_. *Asci* clavate, *Aspicilia*-type. *Ascospores* rarely developed, almost spherical (Figure 8B), colorless, unicellular, 19.0–20.5–22.0 × 14.0–16–17 µm, length/width ratio 1.2–1.3 (n = 5). *Pycnidia* black, immersed in the thallus, *Pycnospores* bacilliform to filiform, straight to slightly curved (Figure 8C), 10.1–10.4–11.0 × 0.3–0.4 µm (n = 10).

**Chemistry:** no metabolites traced by HPTLC in solvent systems A, B, C. 

**Habitat and distribution:** in the splash water zone of permanent and temporarily dry sun-exposed stretches of streams. Some of the specimens are partially overgrown by *Lobothallia hydrocharis*, with cracked-areotated brown *Verrucaria* sp., *Kuettlingeria atroflava* (Turner) I.V. Frolov, Vondrák & Arup., *Dermatocarpon luridum* (With.) J.R. Laundon. *Circinaria ochraceae* sp. nov. is host to *Placopyrenium pseudocinereoatrum* sp. nov. so far only known from the island of Sardinia, where it was found at high elevations (810 and 1030 m) in the provinces of Sassari and Osristano.

**Additional specimens studied:** Additional specimens studied: ibid, specimen infected by *Placopyrenium pseudocinereoatrum*, SMNS-STU-F-0002808; Oristano Prov., Marghine mountain chain, Nuraghe Ortachis, Badde Salighe; N40.347791 E8.906399; 1030 m; July 2014; Nascimbene leg.; on siliceous rock, in the splash zone; BOLO-Herb. Nascimbene 4855 (with *Lobothallia hydrocharis*), SMNS-STU-F-0001655 (STU), SMNS-STU-F-0002799 (STU).

**Notes:** Morphologically and ecologically, the new species also resembles *A. laevatoides* (H. Magn.) Oxner from which it differs by a deeply rimose-areolated thallus with a distinctive white medulla, larger spores and the yellow cortical pigment, which is partially soluble in KOH. The spherical ascospores and bacilliform pycnospores support the placement of this new species in the genus *Circinaria*. ITS data suggest a placement close to the grey-colored fruticose *Circinaria mansourii* (Supplementary Materials Figure S1) from dry steppe soils, which is only known from sterile specimens. Morphologically and ecologically, *Circinaria ochracea* differs from *C. mansourii* by the orange, crustose thallus with richly fertile areoles, absence of fatty acids as detectable metabolites in HPTLC, and its occurrence on rocks in periodically inundated sites. However, a comprehensive genetic study including additional field sampling and multiple loci analyses would be required to better support the genetic isolation of *C. ochracea*.

***Catillaria chalybeia* subsp. *chalybeia* (Borrer) A. Massal.**

Nuoro Prov.; Rio Calaresu, near Cantoniera Pira e Onni; N40.023713 E9.389944; 880 m; July 2014; Nascimbene leg.; on siliceous rock, in the splash zone; BOLO-Herb. Nascimbene 4826, SMNS-STU-F-0001995 (STU).

A widespread species is often more or less shaded and humid to wet places on siliceous rocks. It is a common component of freshwater lichen communities in Western and Central Europe, including the Italian Alps, but so far, had not been reported from Mediterranean regions in this habitat. Although *C. chalybeia* ssp. *chloropoliza* is known from Sardinia [16], and in NW Europe, this is the more frequent subspecies in freshwater habitats [37], we found only the ssp. *chalybeia* in our collections from Sardinian streams.

***Dermatocarpon luridum* (With.) J.R. Laundon**

Nuoro Prov.; Rio Calaresu, near Cantoniera Pira e Onni; N40.023713 E9.389944; 880 m; July 2014; Nascimbene leg.; on siliceous rock, in the splash zone; SMNS-STU-F-0001656 (STU).

Sassari Prov.; Burgos; N40.37795 E8.941887; 820 m; July 2014; Nascimbene leg.; on siliceous rock, in the splash zone; SMNS-STU-F-0002798 (STU).

Oristano Prov., Marghine mountain chain, Nuraghe Ortachis, Badde Salighe; N40.347791 E8.906399; 1030 m; July 2014; Nascimbene leg.; on siliceous rock, in the splash zone; SMNS-STU-F-0002810 (STU).

Characteristic and widespread species of hard and stable rocks in the splash water zone of streams and rivers and in seepages of cliffs with alkaline to slightly acidic water.

***Dermatocarpon miniatum* (L.) W. Mann**

Sassari Prov.; Burgos; N40.37795 E8.941887; 820 m; July 2014; Nascimbene leg.; on siliceous rock, in the splash zone; SMNS-STU-F-0002801 (STU).

*Dermatocarpon miniatum* is a polymorphic species complex in need of revision [45]. The Sardinian collection has typical characteristics of collections from siliceous rocks, with large unilobate thalli and medium-sized ascospores (14–15 µm). Elsewhere in Italy, such forms are found mostly on slightly seeping rocks, but temporarily inundated populations from hard siliceous outcrops on river banks are known from Central Europe, e.g., from a lowland river in the Czech Republic [44], while at high (subalpine) elevations such lineages in freshwater streams and springs are replaced by *D. arnoldianum* auct. medieur. [15,46].

***Hydropunctaria rheithrophila* (Zschacke) C. Keller, Gueidan & Thüs**

Nuoro Prov.; Rio Calaresu, near Cantoniera Pira e Onni; N40.023713 E9.389944; 880 m; July 2014; Nascimbene leg.; on siliceous rock, in the submerged zone; BOLO-Herb. Nascimbene 2643 and 2644; ibid. SMNS-STU-F-0001996 (STU).

This is a characteristic species of usually permanently submerged rocks, but very few records exist from the Mediterranean region.

New to Sardinia.

***Ionaspis chrysophana* (Körb.) Stein**

Nuoro Prov.; Rio Calaresu, near Cantoniera Pira e Onni; N40.023713 E9.389944; 880 m; July 2010; Nascimbene leg.; on siliceous rock, in the splash zone; BOLO-Herb. Nascimbene 4826; duplicate: SMNS-STU-F-0001995 (STU).

A characteristic species on siliceous rocks in wet flushes and in the splash water zone of streams in arctic-alpine regions of the Northern Hemisphere. It rarely occurs in the (high-)montane zones of Central Europe, e.g., in France [18] and Germany [21], where it is regarded to be represented by highly threatened relict populations. Several thalli were found on the same pieces of rock as our collection of *Ionaspis odora* (Schaer.) Stein.

New to the Mediterranean region.

***Ionaspis odora* (Schaer.) Stein**

Nuoro Prov.; Rio Calaresu, near Cantoniera Pira e Onni; N40.023713 E9.389944; 880 m; July 2010; Nascimbene leg.; on siliceous rock, in the splash zone; BOLO-Herb. Nascimbene 4826; duplicate = SMNS-STU-F-0001995 (STU).

This is an upland species in Italy, previously known only from the Alps and the Northern Apennines. It is very rare in the Mediterranean region, known to occur, e.g., in streams at high elevations on Corsica [18]. Central European populations outside of the Alps are declining [42]. Several thalli were found on the same pieces of rock as our collection of *I. chryosphana* (Körb.) Stein.

New to Sardinia.

***Kuettlingeria atroflava* (Turner) I.V. Frolov, Vondrák & Arup**

Nuoro Prov.; Rio Calaresu, near Cantoniera Pira e Onni; N40.023713 E9.389944; 880 m; July 2014; Nascimbene leg.; on siliceous rock, in the splash zone; BOLO-Herb. Nascimbene 2646.

Sassari Province: Burgos; N40.37795 E8.941887; 820 m; July 2014; Nascimbene leg.; on siliceous rock, in the splash zone; SMNS-STU-F-0002932 (STU), ibid. overgrowing *Aspicilia aquatica*; BOLO-Herb. Nascimbene 7173. 

This widespread lichen is a rather constant element in the collections of freshwater lichens from Sardinia, often in small quantities on rock fragments with other lichen species (particularly *Aspicilia* spp. and areolate-cracked *Verrucaria* spp., but not on *Circinaria ochracea* sp. nov.), frequently growing on their thalli. The exact type of interactions with the host lichens requires further studies, but we consider this species as a potentially facultative lichenicolous lichen. ITS data from one of the Sardinian samples are identical to those of a specimen from the Czech Republic but with minor differences with samples from other European localities.

***Lathagrium fuscovirens* (With.) Otálora, P.M. Jørg. & Wedin**

Carbonia-Iglesias Prov., Domus Novas, Duchessa, Monti Mannu; N39.392102 E8.669081; 305 m; July 2014; Nascimbene leg.; on siliceous rock, in the splash zone; SMNS-STU-F-0002801 (STU).

A widespread and common species, which is occasionally found in subaquatic communities, e.g., on the banks of streams and rivers [47] but not restricted to this habitat.

***Lepra corallina* (L.) Hafellner**

Oristano Prov., Marghine mountain chain, Nuraghe Ortachis, Badde Salighe; N40.347791 E8.906399; 1030 m; 11/03/2014; Nascimbene leg.; on siliceous rock, in the splash zone; SMNS-STU-F-0002809 (STU).

A rather common and widespread species from siliceous rocks but previously not known as a part of freshwater lichen communities.

***Lobothallia hydrocharis* (Poelt & Nimis) Sohrabi & Nimis**

Specimens studied: Nuoro Prov.; Rio Calaresu, near Cantoniera Pira e Onni; N40.023713 E9.389944; 880 m; July 2010; Nascimbene leg.; on siliceous rock, in the splash zone; BOLO-Herb. Nascimbene 2642. Ibid.; Rio Calaresu, near Cantoniera Pira e Onni; N40.023713 E9.389944; 860 m; July 2014; Nascimbene leg.; on siliceous rock, in the splash zone; BOLO-Herb. Nascimbene 4852. Carbonia-Iglesias Prov., Domus Novas, Duchessa, Monti Mannu; N39.379871 E14.607985; 380 m; July 2014; Nascimbene leg.; on siliceous rock, in the splash zone; BOLO-Herb. Nascimbene 4854. Oristano Prov., Marghine mountain chain, Nuraghe Ortachis, Badde Salighe; N40.347791 E8.906399; 1030 m; July 2014; Nascimbene leg.; on siliceous rock, in the splash zone; BOLO-Herb. Nascimbene 4855 and 4856, SMNS-STU-F-0002799 (STU). Sassari Prov.; Burgos; N40.37795 E8.941887; 820 m; July 2014; Nascimbene leg.; on siliceous rock, in the splash zone; BOLO-Herb. Nascimbene 4857; ibid. SMNS-STU-F-0002807 (STU); ibid. SMNS-STU-F-0002807 (STU).

Our records considerably enlarge the known distribution of this species on Sardinia, where the species appears to be widespread but rare at high elevations [22]. It is now known from mountain chains with siliceous bedrock in four of the eight provinces of Sardinia. At most of these sites *Lobothallia hydrocharis* is the dominant species in the splashwater zone (Figure 2D).

We found it growing in isolated patches, as well as epilichenic on other crustose lichens, particularly *Circinaria ochracea* sp. nov., and *Verrucaria mediterranea* sp. nov. (Figure 7). Epilichenic growth of this species was reported before [16], but the host in these earlier observations was identified as *V. anziani* Garov. Unfortunately, no vouchers could be traced in GZU to confirm this identification, and no other collections of *V. anziana* could be found. We therefore consider the possibility that the host for these old observations was, in fact, *V. mediterranea* as well, such as in our recent collections. *Lobothallia hydrocharis* is a characteristic element of temporary and permanent streams and small rivers on Sardinia.

***Placopyrenium pseudocineroatrum* Thüs & Nascimbene sp. nov. MB 847529**

Holotype: Sassari Prov.; Burgos; N40.37795 E8.941887; 820 m; July 2014; Nascimbene leg.; on siliceous rock, in the splash zone parasitic on *Circinaria ochracea*; SMNS-STU-F-0002927 (STU), Isotype: SMNS-STU-F-0002808 (STU).

**Diagnosis:** dark brown-grey and deeply cracked crust with immersed perithecia lacking an involucrellum. Similar to *Placopyrenium cinereoatrum* (Degel.) Orange but with (on average) slightly shorter ascospores, initially on semi-aquatic *Circinaria-* instead of *Staurothele*-species as hosts, and with different ITS sequences.

**Figure 9:**
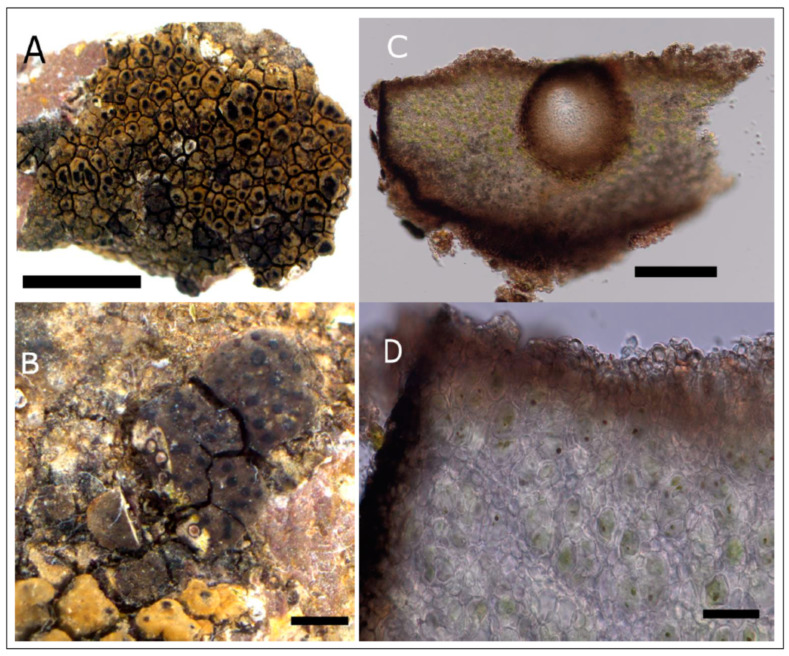
(**A**) Holotype of *Placopyrenium pseudocinereoatrum* with young perithecia (=SMNS-STU-F-0002927) on *Circinaria ochracea* (=SMNS-STU-F0002797). (**B**) Isotype specimen with sectioned mature perithecia, SMNS-STU-F-0002808. (**C**) Section with young perithecium in subsquamulose thallus part. (**D**) Thallussection with pseudocortex and photobiont layer from the holotype. (**D**) Scale bars in (**A**) = 5 mm, in (B) = 1 mm, (**C**) = 0.2 mm, (**D**) = 0.02 mm.

**Description**: *Prothallus* not seen. Parasitic forms on *Circinaria ochracea* at first visible as a brown-grey shade on the host (Figure 9A), with increased crack density and blackened margins of the areoles, eventually developing a thick crustose to subsquamulose thallus with almost fully immersed spherical perithecia (Figure 9B), marginal areoles in the independent form slightly elongated. *Thallus* up to 460 µm high, locally with more or less developed thin epinecral layer of dead but not collapsed cells on top of app. 10 µm pseudocortex with brownish pigment, fungal cells in cortex and photobiont layer rounded, app. 4 µm diam. (Figure 9D). *Photobiont layer* app. 200 µm high, photobiont cells in clusters, cells app. 10 µm diam. *Medulla* in the parasitic form colorless to brownish in the lowermost parts (Figure 9C), brown-black in the independently growing form. *Perithecia* dispersed to clustered, without involucrellum, starting to develop below the surface of the thallus, eventually emerging with the opening ostiole slightly over the thallus surface. *Exciple* dark black-brown at the top, brown below, up to 240 µm diam., periphyses app. 30 × 2 µm. *Ascospores* 8 per ascus, 11.0–17.5–21.0 × 6.5–7.5–9.5 µm, length/width ratio 1.9–2.2–2.3 (n = 23), colorless, without perispore, occasionally with spores of distinctly different size classes in the same perithecium. *Pycnidia* tips slightly projecting over the thallus, with black pigmentation at the top, colorless below, *Dermatocarpon*-type, pycnospores rod-shaped, straight or slightly curved, app. 4–7 × 1 µm, colorless (seen only in the non-parasitic form on the isotype).

**Habitat and Distribution:** lichenicolous on *Circinaria ochracea* sp. nov. when young, older thalli also without visible host thallus remains; growing in the splash water zone of unshaded streams with permanent or temporary waterflow, together with semi-aquatic lichens, such as *Dermatocarpon luridum*, *Lobothallia hydrocharis* and its host *Circinaria ochracea*. Only known from two provinces on the island of Sardinia.

**Additonal specimen studied:** Oristano Prov., Marghine mountain chain, Nuraghe Ortachis, Badde Salighe; N40.347791 E8.906399; 1030 m; 11/03/2014; Nascimbene leg.; on siliceous rock, in the splash zone, parasitic and with independently living thalli among other lichens, e.g., cracked-areotated brown *Verrucaria* sp., *Lobothallia hydrocharis*, *Kuettlingeria atroflava*, *Dermatocarpon luridum*, BOLO-Herb. Nascimbene 4378.

**Notes:** Our spore measurements are overlapping with the range for *P. cinereoatrum,* but young thalli in our collections are clearly parasitic on *Circinaria ochracea* sp. nov., not on *Staurothele fissa,* the only known host for *P. cinereoatrum* [12]. ITS data confirm that the Sardinian *Placopyrenium* on *Circinaria ochracea* sp. nov. is related to *P. cinereoatrum* but differs substantially in several regions of this marker. The thalli are only faintly pruinose, dark brown-grey, and never as light grey as typical specimens of *P. cinereoatrum*, but there is a wide overlap in color between the two species. The new species differs ecologically by its growth on a host from a different family and possibly furthermore biogeographically in its absence from areas north of the Alps. Sequences of *P. cinereoatrum* from Britain, Finland and the Czech Republic are remarkably homogenous despite the large geographic distances. The Sardinian taxon stands out by its clear separation from the Central and Northern European clade. This pattern—together with its different hosts—supports the assumption that the Sardinian lichen is indeed an independent species. There are no other *Placopyrenium* species known to occur on *Circinaria* in subaquatic habitats, but freshwater *Aspicilia* thalli are hosts for *Placopyrenium formosum* [12]. The spores of the latter species are much larger, it is characterized by the presence of a brown prothallus, and its ITS sequences differ largely from those of the new species and *P. cinereoatrum* (Figure 4). Morphologically the independently growing form of the new species has some resemblance to *P. trachyticum* (Hazsl.) Breuss, which is only known as an independently growing (not parasitic) lichen of drier places, also differing in the more elongated and narrower ascospores (5–7.5 um).

***Placopyrenium bucekii* (Nádv. & Servít) Breuss**

Sassari Prov.; Burgos; N40.37795 E8.941887; 820 m; July 2014; Nascimbene leg.; on siliceous rock, in the splash zone; SMNS-STU-F-0002794 (STU); ibid. SMNS-STU-F-0002804 (STU).

One of the thalli in the two collections appears to be parasitic on a living *Verrucaria* sp. The other one consists of apparently old and very thick, independently growing thalli without visible remains of a possible host lichen. Dead remains of a *Verrucaria* species are only sparsely visible in the direct vicinity of the margins of one of these thalli. We found *P. buceckii* in the splash water zone together with other lichens typical for this habitat. A parasitic lifestyle with crustose Verrucariaceae as hosts were reported for this species from France [48]. It had been collected on Sardinia before in the provinces of Nuoro, Cagliari and Sassari [16], but not in the splash water zone of streams. Elsewhere in Italy, it is mostly found on seeping rocks.

ITS data of the Sardinian lichen are identical to those obtained from France.

***Rinodina fimbriata* Körb.**

Sassari Prov.; Burgos; N40.37795 E8.941887; 820 m; July 2014; Nascimbene leg.; on siliceous rock, in the splash zone; SMNS-STU-F-0002792, SMNS-STU-F-0002800 (STU).

In Europe, this is a widespread but rare species with a scattered distribution, mostly in oceanic parts of the continent and usually in the splash water zone of more or less shaded streams [49,50,51]. It has been recorded from Sardinia before at a single site in the northern province of Nuoro in 1985 [16]. On the rock fragment that we collected in Burgos, this species was associated with typical lichens of the splash water zone of Sardinian mountain streams (*Verrucaria* cf. *cernaensis, Lobothallia hydrocharis* and *Circinaria ochracea*).

***Squamulea subsoluta* (Nyl.) Arup, Søchting & Frödén**

Sassari Prov.; Burgos; N40.37795 E8.941887; 820 m; July 2014; Nascimbene leg.; on siliceous rock, in the splash zone; SMNS-STU-F-0002793 (STU).

This species is widespread in sun-exposed sites, elsewhere also found in association with species from seeping rocks (e.g., *Peltula euploca* (Ach.) Poelt ex Pišút) but so far not reported as a component of freshwater lichen communities in streams.

***Staurothele fissa* (Taylor) Zwackh**

Oristano Prov., Marghine mountain chain, Nuraghe Ortachis, Badde Salighe; N40.347791 E8.906399; 1030 m; 11/03/2014; Nascimbene leg.; on siliceous rock, in the splash zone; BOLO-Herb. Nascimbene 4376, SMNS-STU-F-0002805 (STU).

This is a characteristic species of the splashwater zone in clean streams and rivers with large rocks, boulders or outcrops in the riverbed, mostly in cool areas. Our material corresponds to *S. fissa* s. str. with brown spores. *Staurothele hazslinszkyi* (Körb.) J. Steiner is treated by some authors as a younger synonym of *S. fissa*. Previous records of *S. hazslinskyi* from Sardinia [16] refer to specimens from drier habitats with persistently hyaline spores. If the two taxa are synonymous remains to be clarified.

***Verrucaria aquatilis* Mudd**

Nuoro Prov.; Urzulei, Gennargentu National Park, along the road n° 125 (“orientale sarda”) near Genna Sarbene, Riu Pirughedda; N40.103450 E9.564714; 750 m; July 2009; Nascimbene leg.; on granitic rock, in the submerged zone; BOLO-Herb. Nascimbene 2272. Nuoro Prov.; Rio Calaresu, near Cantoniera Pira e Onni; N40.023713 E9.389944; 880 m; July 2010; Nascimbene leg.; on siliceous rock, in the submerged zone; BOLO-Herb. Nascimbene 2645, SMNS-STU-F-0002144 (STU).

*Verrucaria aquatilis* is a quick colonizer of submerged rocks and pebbles with a wide distribution in humid parts of the European continent, with very scattered records from the Mediterranean region.

New to Sardinia.

***Verrucaria* cf. *cernaensis* Zschacke**

Nuoro Prov.; Rio Calaresu, near Cantoniera Pira e Onni; N40.023713 E9.389944; 880 m; July 2010; Nascimbene leg.; on siliceous rock, in the submerged zone; SMNS-STU-F-0002791 (STU); Sassari Prov.; Burgos; N40.37795 E8.941887; 820 m; July 2014; Nascimbene leg.; on siliceous rock, in the splash zone with *Rinodina fimbriata,* SMNS-STU-F-0002800 (STU).

The recent collections from Sardinia are from a temporarily inundated site. The spores are 20–22.5 × 7.5 µm, perithecia fully immersed and thallus smooth and continuous in young parts, becoming cracked-areolate towards the centre. A dark basal layer is absent. With the key for species from freshwater habitats in [21] our specimen is identified as *V. cernaensis*. It differs from the newly described *V. mediterranea* sp. nov. by completely immersed perithecia, larger spores, a thicker thallus and the lack of a light grey color component. Thallus color and structure, shape of the involucrellum and the immersed perithecia are also similar to the type of *V. endocarpoides* Servít from drier habitats, but the periphyses in our collection are much thinner and the spores are smaller. The morphologically variable *Verrucaria viridula* (Schrad.) Ach. can appear similar in the field but has larger spores with a much lower length/width ratio and a reduced or absent involucrellum.

We have obtained an ITS sequence from the specimen from Burgos, but it did not match sequences of *V. cernaensis* on GenBank, and it remained uncertain if it represents a parasitic inhabitant of the lichen thallus or the main mycobiont.

There are earlier collections of *V.* cf. *cernaensis* from Sardinia by [16] from non-aquatic sites in Nuoro Province, Gennargentu Massif, close to the Rio Aratu (GZU 80–86)) and from a stream bed in Catena del Marghine N of Punta Palai (GZU P1–85). Although these older samples had been annotated as *V.* cf. *cernaensis* by H. Ullrich, he also indicated that an alternative identification as *V. buellioides* Servít would be possible based on the key by [52]. *Verrucaria buellioides* is a poorly known species in need of revision [53].

***Verrucaria collematodes* Garov. (=*V.nigrescens* s.lat.?)**

Nuoro Prov., Genna Sarbene, Riu Pirughedda, 750 m, July 2009, on periodically inundated granite. J. Nascimbene leg.; BOLO-Herb. Nascimbene 2274, 2275, 2276.

The collections from Sardinia are characterized by brown to greyish brown thalli with immersed to slightly projecting perithecia. The areoles swell up considerably when wet and large areas of the thallus have a black basal layer merging with the thick involucrella of the perithecia. This poorly known species possibly belongs to *V. nigrescens* s.lat [21].

New to Sardinia.

***Verrucaria devensis* Orange**

Oristano Prov., Marghine mountain chain, Nuraghe Ortachis, Badde Salighe; N40.347791 E8.906399; 1030 m; 11/03/2014; Nascimbene leg.; on siliceous rock, in the submerged zone; BOLO-Herb. Nascimbene 4377, duplicate: SMNS-STU-F-0002806 (STU).

**Figure 10:**
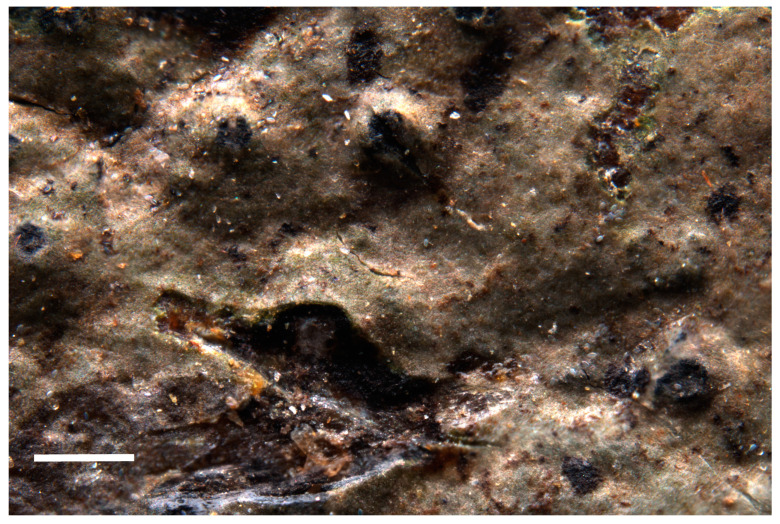
Specimen of *Verrucaria devensis* from Sardinia with smooth and almost uncracked thallus, SMNS-STU-F0002806. Scale bar = 0.5 mm.

The specimen from Sardinia is rather thin, with a brownish green color (Figure 10), a continuous thallus, which is mostly free of cracks and an almost subgelatinous appearance when wet. Due to this unusual morphology, it was at first confused with *Verrucaria funckii* (Spreng.) Zahlbr. The photobiont cells are not arranged in vertical columns and the thallus does not turn completely transparent when wet, even in areas where the black basal layer is missing. *Verrucaria devensis* is known from more or less shaded terrestrial but also more exposed sites when temporarily inundated. *Verrucaria funckii* instead is best developed in permanently inundated micro-habitats and only reaches out into the lower splash water zone. Despite the morphological differences and the large distance between the collection sites, the ITS signature of the Italian specimen and the one of the type material of *V. devensis* from the British Isles are almost identical (99.66% over a length of 764 bp).

New to Italy and Southern Europe.

***Verrucaria mediterranea* Thüs & Nascimbene sp. nov. MB#847531**

Holotype: Carbonia-Iglesias Prov., Domus Novas, Duchessa, Montimannu; N39.392102 E8.669081; 305 m; July 2014; Nascimbene leg.; on siliceous rock, in the splash zone; SMNS-STU-F-0001654 (STU). Isotype: ibit, BOLO-Herb. Nascimbene 8041.

**Diagnosis:** thallus light-brown to light-grey with perithecia conical, reaching down to the thallus base, similar to those in *Verrucaria fuscoatroides* Servít and *V. nigrofusca* Servít, but with shorter and narrower spores.

**Figure 11:**
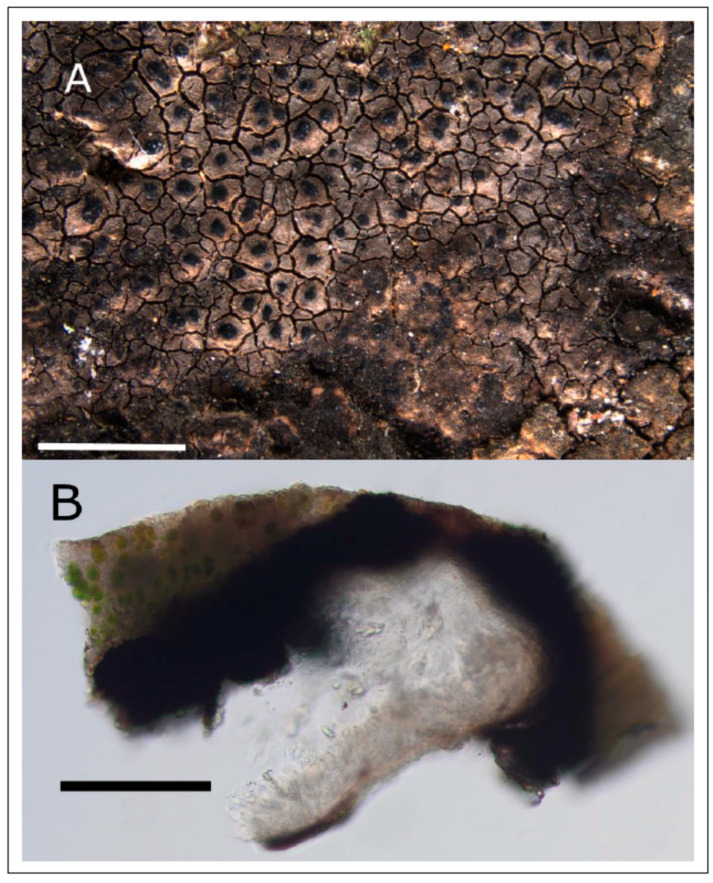
Holotype of *Verrucaria mediterranea* sp. nov., SMNS-STU-F-0001654 (STU). (**A**) Outer morphology. (**B**) Section of a perithecium with ascospores, and surrounding thallus. Scale bars in (**A**) = 1 mm, in (**B**) = 100 µm.

**Description:** thallus light-brown to light-grey, 0.15 mm high, rimose to areolate, the areoles ca. 0.3 mm wide (Figure 11A). *Pseudocortex* with brownish pigment, in some spots with black basal layer, others without distinguishable medulla. *Photobiont* a green Chlorophyta algae, with coccoid to elongated cells, mostly with more or less spherical cells app. 8–10 μm diam., some elongated larger cells interspersed with a size up to 20 × 10 μm. *Perithecia* ⅔ to fully immersed in the thallus, with a distinct involucrellum reaching down to the basis of the perithecium, attached to the exciple but thickened at its base, forming a conical structure (Figure 11B). *Periphyses* long (up to 50 μm) and thin (1.5–1.8 μm) [n = 15]. *Exciple* dark brown from top to bottom. *Asci* clavate ca. 64 × 19 μm. *Ascospores* 15–18.9–21 × 6.0–7.4–8.4 μm, length/width ratio 1.9–2.4–3.3 [n = 37].

**Habitat and Distribution:** splash water zone of montane streams. We found this species at three different sites, each in a different province on the island of Sardinia from 300 to over 1000 m, where it formed extensive colonies together with typical freshwater species, e.g., *Aspicilia aquatilis*, *Circinaria ochracea,* etc. Some specimens are overgrown by *Dermatocarpon luridum* and/or host to epilichenic forms of *Kuettlingeria atroflava* and *Lobothallia hydrocharis*.

**Additional specimens studied:** Oristano Prov., Marghine mountain chain, Nuraghe Ortachis, Badde Salighe; N40.347791 E8.906399; 1030 m; 11/03/2014; Nascimbene leg.; on siliceous rock, in the splash zone; SMNS-STU-F-0001655 (STU).

Burgos Prov., N40.37795 E8.941887; 820 m; on siliceous rock, in the splash water zone; SMNS-STU-F-0002795 (STU).

**Notes:** The thallus structure and perithecia are similar to those described for *V. fuscoatroides* Servít and *V. nigrofusca* Servít [50], but the spores of the new species are shorter and narrower. *Verrucaria fuscoatroides* was reported from temporarily inundated sites (Austria: [53]) and may be widespread but under-recorded [49]; due to its more frequent occurrence in drier habitats, it does not appear in any of the European keys specifically dedicated to freshwater lichens. *Verrucaria nigrofusca* is a poorly known taxon of similar morphology but with a slightly smaller exciple and spores, the latter however still larger than those of *V. mediterranea* sp. nov. *Verrucaria atroviridula* Zschacke, which is only known from its type collection [54,55], is also similar but with much more prominent perithecia, wider spores, and an involucrellum, which spreads and separates from the exciple in its lowermost part. None of these species develops a black basal layer or brownish areas in the medulla. *Verrucaria mediterranea* sp. nov. would key out as *V. cernaensis* in the keys of [6,49]. It differs by usually thinner thalli, often more prominent perithecia and a light-grey color, but the differences are subtle, and ITS barcoding may be necessary for safe identification. BLAST search against GenBank sequences suggests an affinity to taxa from the *Endocarpon* clade sensu [3]. In our ITS-genetree, the sequences of the new species are not nested within the *V. nigrescens*/*czernaesis* group but more closely related to “*Verrucaria* sp. 10” and to the recently described *V. tenuispora* Vondrák and Thüs from the Czech Republic [44] (Figure 4), a species which differs from the Sardinian species by its thallus areoles, which are uneven, eventually becoming ± squamulose thallus with granular margins and by much more elongated ascospores. Species of the “*Verrucaria tenuispora*-group” [44] and *V. mediterranea* share the trait of the occurrence of photobiont cells in the thallus, which can reach a size of up to 20 μm when fully mature, almost twice the size of the photobiont cells in the lichen thalli of most other Verrucariaceae.

***Verrucaria pseudovirescens* Servít**

Nuoro Prov., Rio Pardu, May 1986, J. Poelt leg., GZU 80–86 (GZU).

An old collection with a brown, areolated thallus lacking a black basal layer was provisionally identified by H. Ullrich as *Verrucaria* cf. *cataleptoides* [16]. Re-examination of the collection revealed that the involucrellum in this specimen is spreading and the thallus is far too thin (only up to 25 µm) for *V. cataleptoides*. Because this was the only collection, this name should be removed from the checklist of lichens of Sardinia. Its morphology fits with the description of *V. pseudovirescens*, except that the latter species is usually found on calcareous rocks and often contains lime crystals in the thallus, while the Sardinian lichen grows on siliceous rocks and lacks mineral inclusions. The species has been described from the Verona province [56], but it has long been neglected and was only recently recorded again from Russia [57], Austria [58], Switzerland [59] and Germany [60].

New to Sardinia.

***Verrucaria ruderum* DC.**

Sassari Prov.; Burgos; N40.37795 E8.941887; 820 m; July 2014; Nascimbene leg.; on siliceous rock, in the splash zone of a stream, partially overgrown by *Lobothallia hydrocharis*; BOLO-Herb. Nascimbene 4375.

**Figure 12:**
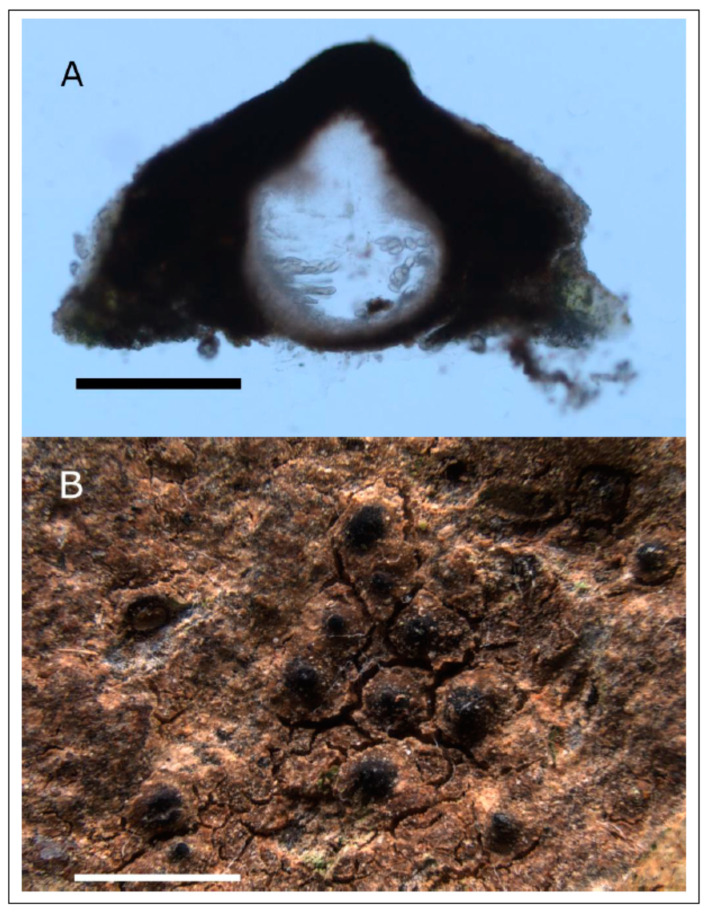
*Verrucaria ruderum* from Sardinia BOLO-Herb. Nascimbene 4375. (**A**) Section of a perithecium with elongated ostiolum and surrounding thallus. (**B**) Outer morphology. Scale bars in (**A**) = 1 mm, in (**B**) = 100 µm.

This is a rarely recorded but possibly overlooked species [49] and its circumscription remains poorly understood [21]. Morphologically the Italian collection is identified as *V. ruderum* based on the ostiolum forming a distinctively projecting “neck” (Figure 12A), spore size and shape (28–34 × 17–23 µm), absence of a dark medulla and the cracked thallus (Figure 12B). ITS data place our specimen in the *V. viridula* agg. with a specimen from the British Isles (Figure 4).

To our knowledge, neither *V. ruderum* nor *V. viridula* have been reported from temporarily inundated habitats before.

### 3.2. Comparison with Freshwater Lichen Biota under Different Bioclimatic Conditions

**Figure 13:**
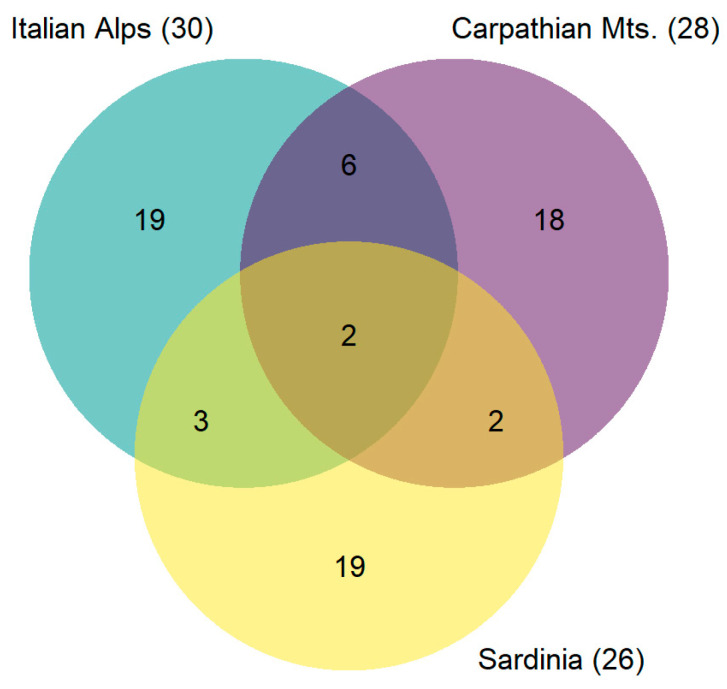
Venn diagram of species occurrences in freshwater courses of Sardinia and in the reference sites in the Italian Alps and Eastern Carpathian Mts. For each locality, the number of unique species is indicated (where the circle does not overlap), as well as that of the species shared among sites (overlapping parts).

In terms of taxonomic composition, the freshwater lichen biota associated with *Lobothallia hydrocharys* in Sardinia showed low overlap with species lists for freshwater habitats at the two reference sites in the Alps and Carpathians (Figure 13), sharing less than 20% of the species but with 73% of the species that are unique (63% and 64% for the Alps and Carpathians, respectively). Only *Dermatocarpon luridum* and *Hydropuntaria rheitrophila* were shared among all three areas. *Dermatocarpon miniatum*, *Ionaspis odora*, and *Staurothele fissa* were shared between Sardina and the Alps, while *Aspicilia aquatica* and *Verrucaria cernaensis* were shared between Sardina and the Carpathians. In contrast, there were eight species shared between the Alps and the Carpathians, including *Bacidina inundata*, *Thelidium minutulum*, *T. submethorium*, *Verrucaria funckii*, and *V. margacea*. In terms of species traits, in Sardinia we found a low percentage of subgelatinous species compared to the areas in the Alps (double value) and Carpathians (Table 2). In contrast, the incidence of gelatinous species (foliose Collemataceae) and cyanolichens, in general, was somehow comparable between the studied areas in the Alps and Sardinia, while lichens with these traits were not recorded at all in the study sites of the Carpathian area.

## 4. Discussion

The exploration of freshwater lichens of Sardinia resulted in a surprisingly species-rich lichen biota associated with the regional flagship species *Lobothallia hydrocharis*. The inclusion of the latter in the genus *Lobothallia*, as a sister species in relation to *L. radiosa* was supported by molecular data. The survey also yielded three species new to science in the genera *Verrucaria*, *Placopyrenium*, and *Circinaria*, descriptions of the new species being supplemented by ITS barcoding data. Additional new species, e.g., from the family Megasporaceae, are likely included in our material, but we preferred to avoid a formal description until more data are available.

The high yield of new species indicates that Mediterranean watercourses should be specifically targeted in future lichen surveys to fill knowledge gaps on lichen biodiversity in the Mediterranean basin. We also recognize the need for an acceleration of efforts to re-collect and sequence poorly known species, particularly from the families Verrucariaceae and Megasporaceae to minimize the risk of duplicate descriptions for species encountered in modern surveys and to facilitate accurate identification of specimens based on DNA-barcoding approaches.

The collections at hand were already rather old (8–13 years) and kept at room temperature before DNA-extraction could be performed. This may explain why only ITS performed well enough in PCR to aid in the taxonomic treatment of taxa, while attempts to amplify other markers failed. This observation supports the consideration by Orange [61] that collectors who visit underexplored regions of the world should be encouraged to place at least some fruiting bodies of each taxon in freezers if immediate DNA extraction is not an option.

Our results support the view that the lichen biota of Sardinian watercourses may have peculiar taxonomic composition and specific traits if compared with similar environments under different bioclimatic conditions, as for example in the Alps and the Carpathians. This is indicated by relatively low taxonomic overlap and differences in the incidence of some traits. In particular, the lichen biota of Sardinian watercourses seems to be poor in subgelatinous lichens, which can be likely related to the more stressful hydration patterns in Mediterranean watercourses where lichens have to cope with periods of abundant and impetuous water flow alternating with long periods of complete dryness and warm to hot temperatures.

Furthermore, the counterintuitive placement of *L. hydrocharis* in the ITS genetree of the genus, indicating a stronger relationship with the terrestrial *L. radiosa*, which often occurs in arid and sun-exposed situations, and *L. recedens*, another species from exposed sites and only short periods of hydration [62], than with *L. melanaspis* (Ach.) Hafellner, a typical freshwater lichen along alpine siliceous watercourses [47], may reflect peculiar species traits that determine a trade-off between tolerance to inundation, severe desiccation, and overheating while being in the hydrated condition.

Interestingly, the thallus of *L. hydrocharis* has longitudinal cracks not known in other species of this genus [16], which may be interpreted as an adaptation to minimize physical stress from rapid swelling or shrinking of the thallus during periods of quick and severe changes in hydration conditions. In the splashwater, or only temporarily inundated zones of Sardinian watercourses, *L. hydrocharis* is typically associated with Megasporaceae, Teloschistaceae (mostly *Kuettlingeria atroflava*), and Verrucariaceae, most of them also with heavily cracked or areolate and dark-colored to grey thalli, as in the case of the new species *Verrucaria mediterranea*.

In contrast, only two subgelatinous specialists of the family Verrucariaceae (*Verrucaria aquatilis*, *Hydropunctaria rheithrophila*) were found in the submerged zone, where *L. hydrocharis* rarely occurs. Both of them have small perithecia and ascospores, traits that have been associated with a pioneer life strategy [63]. Slow colonizers, instead, appear to be mostly absent, which could indicate that even the subgelatinous specialists in the submerged zone repeatedly colonize and survive in years with sufficient rainfall and permanence of waterflow during summer but disappear in years of prolonged drought. Such an extent of flexibility may be more difficult for other Verrucariaceae with a subgelatinous thallus but with larger ascocarps and slower colonizer life strategies (e.g., *V. funckii*, *V. elaeomelaena* agg.). This hypothesis needs to be tested by more time-series data in long-term systematic field observations.

None of the lichenicolous fungi recorded in our study was included in the checklist of lichenicolous fungi for Sardinia [64]. This may partially be the result of a narrower concept of a lichenicolous lifestyle—excluding most of the species that are also frequently found without contact with remains of a visible host lichen (e.g., excluding *Placopyrenium buceckii*)—but also reflecting the minimal attention paid to freshwater habitats in most previous lichen collections on the island.

## 5. Conclusions

Our study was intended as a pilot project, which can only scratch at the surface of freshwater lichen diversity in Mediterranean streams. Results indicate that under the particular conditions of Mediterranean stream hydrology and climate, functional and taxonomic composition of freshwater lichen communities may be distinct from those of more temperate regions of Europe, being worthy of further research and conservation efforts. Unfortunately, conservation targeted at lichens is still in its infancy, and in Italy, tentative red lists are currently available only for epiphytic and terricolous lichens [65,66]. The lack of these tools for freshwater lichens is mainly related to the scarcity of primary data for reliable species assessment. This situation could be mitigated in the future by including freshwater lichens in already established long-term monitoring sites (e.g., the L-ter network, http://www.lteritalia.it/, accessed 1 November 2022) or in monitoring activities related to the implementation of the EU Water framework directive.

## Figures and Tables

**Table 1:** Specimens for which new ITS barcoding data were obtained and their GenBank-Accession Numbers.

Taxon	Collection Nr.	GenBank-Accession
*Aspicilia* sp. 1	SMNS-STU-F-0002802 (STU)	OQ073917
*Aspicilia* sp. 2	SMNS-STU-F-0002796 (STU)	OQ073920
*Circinaria ochracea*	SMNS-STU-F-0002797 (STU)	OQ073918
*Circinaria ochracea*	JN72085 (BOLO)	OQ073919
*Kuettlingeria atroflava*	SMNS-STU-F-0002932 (STU)	OQ073921
*Lobothallia hydrocharis*	JN72085b (BOLO)	OQ073922
*Lobothallia hydrocharis*	SMNS-STU-F-0002807 (STU)	OQ073923
*Lobothallia hydrocharis*	SMNS-STU-F-0001655 (STU)	OQ073924
*Placopyrenium bucekii*	SMNS-STU-F-0002804 (STU	OQ073925
*Placopyrenium pseudocinereoatrum*	SMNS-STU-F-0002927 (STU)	OQ073926
*Verrucaria devensis*	SMNS-STU-F-0002806 (STU)	OQ073927
*Verrucaria mediterranea*	SMNS-STU-F-0001654 (STU)	OQ073928
*Verrucaria mediterranea*	SMNS-STU-F-0001655 (STU)	OQ073929
*Verrucaria mediterranea*	SMNS-STU-F-0002795 (STU)	OQ073930
*Verrucaria ruderum*	BOLO-Herb. Nascimbene 4375	OQ073931
*Verrucaria* sp.	SMNS-STU-F-0002800 (STU)	OQ073932

**Table 2:** Comparison of selected species traits among similar surveys in mountain areas under different bioclimatic conditions (i.e., Alps and Carpathians vs. Sardinia). The total number of species in each survey is also reported (N species).

	N Species	% Species with Cyanobacteria as Main Photobiont	% Gelatinous Species	% Subgelatinous Species
Italian Alps [41]	30	6.7	3.3	30.0
Eastern Carpathian Mts. [42]	28	0.0	0.0	21.4
Sardinia-this study	26	3.8	3.8	15.4

## Data Availability

Not applicable.

## Supplementary Materials

The following supporting information can be downloaded at: https://www.mdpi.com/article/10.3390/jof9030380/s1, Figure S1: Topology of best phylogenetic RAxML tree based on ITS data of *Circinaria* s.lat., with *Aspicilia* spp. and *Aspicilidea intermedia* as outgroup; Table S1: Voucher information for the specimens that are represented by sequences from GenBank used in this study.
