# Supplementary material for: Freshwater Lichens, Including New Species in the Genera Verrucaria, Placopyrenium and Circinaria, Associated with Lobothallia hydrocharis (Poelt & Nimis) Sohrabi & Nimis from Watercourses of Sardinia
Source: J Fungi (Basel). 2023 Mar 21;9(3):380. doi: 10.3390/jof9030380 (PMC10051252; doi:10.3390/jof9030380)
Supplement: Supplementary file 1 [file Supplementary Materials Figure 1.pdf › Supplementary Materials Figure 1.pdf]

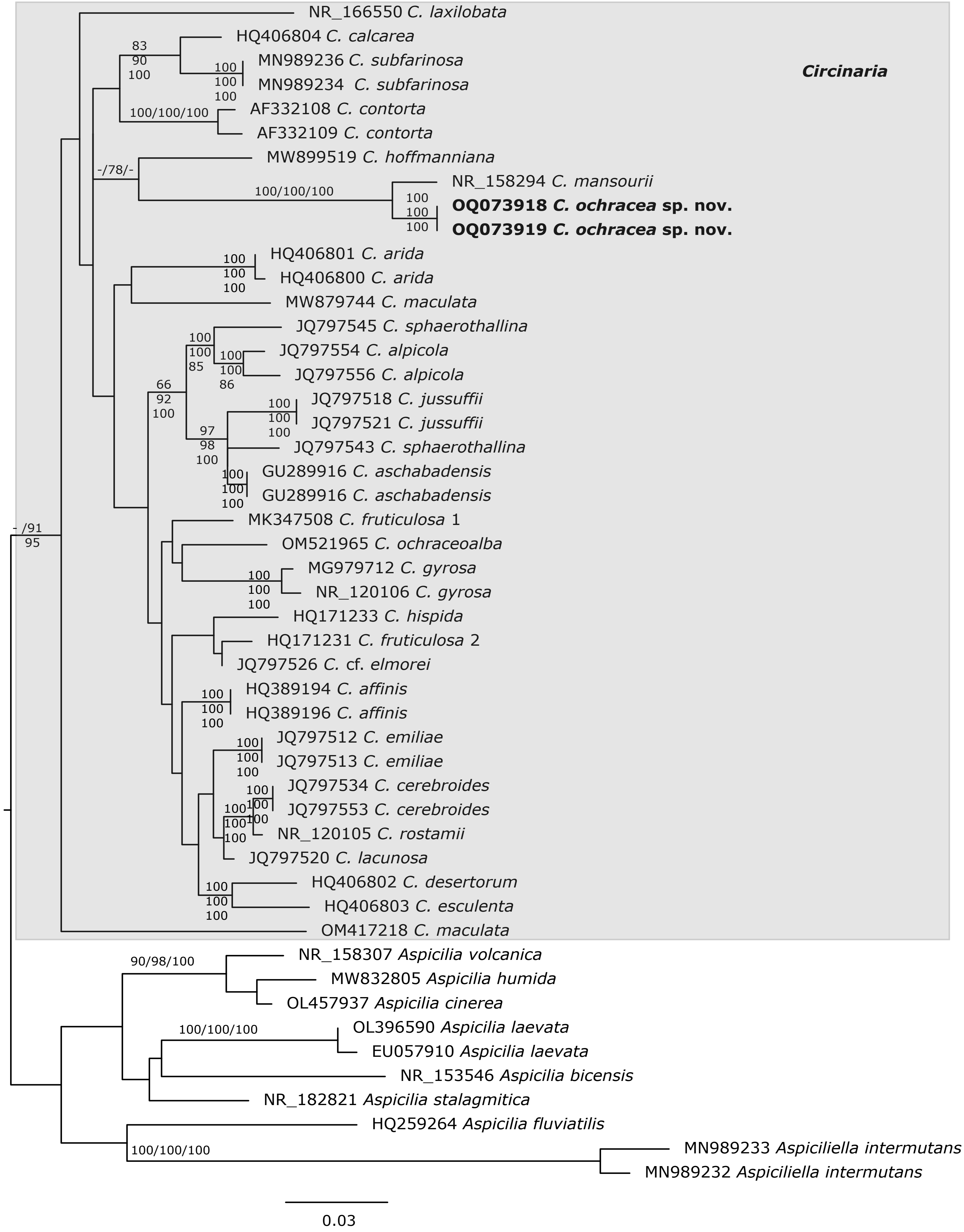


**Supplementary Materials Figure 1.** Topology of best phylogenetic RAxML tree based on ITS data of *Circinaria* s.lat., with *Aspicilia* spp. and *Aspicilidea intermedia* as outgroup. The numbers in each node represent bootstrap support (BS) from RAxML analyses, ultrafast bootstrap support from IQTree- analysis and posterior probability (PP) values from Bayesian Inference of phylogeny. Only BS values ≥ 75% and PP values ≥ 0.95 were plotted on the branches of the tree. GenBank Accession Numbers are given before each taxon name, Sardinian collections are marked in bold.
